# Effects of increased nurses’ workload on quality documentation of patient information at selected Primary Health Care facilities in Vhembe District, Limpopo Province

**DOI:** 10.4102/curationis.v39i1.1545

**Published:** 2016-05-13

**Authors:** Rhulani C. Shihundla, Rachel T. Lebese, Maria S. Maputle

**Affiliations:** 1 Department of Advanced Nursing Science, School of Health Sciences, University of Venda, South Africa

## Abstract

**Background:**

Recording of information on multiple documents increases professional nurses’ responsibilities and workload during working hours. There are multiple registers and books at Primary Health Care (PHC) facilities in which a patient’s information is to be recorded for different services during a visit to a health professional. Antenatal patients coming for the first visit must be recorded in the following documents: tick register; Prevention of Mother-To-Child Transmission (PMTCT) register; consent form for HIV and AIDS testing; HIV Counselling and Testing (HCT) register (if tested positive for HIV and AIDS then this must be recorded in the Antiretroviral Therapy (ART) wellness register); ART file with an accompanying single file, completion of which is time-consuming; tuberculosis (TB) suspects register; blood specimen register; maternity case record book and Basic Antenatal Care (BANC) checklist. Nurses forget to record information in some documents which leads to the omission of important data. Omitting information might lead to mismanagement of patients. Some of the documents have incomplete and inaccurate information. As PHC facilities in Vhembe District render twenty four hour services through a call system, the same nurses are expected to resume duty at 07:00 the following morning. They are expected to work effectively and when tired a nurse may record illegible information which may cause problems when the document is retrieved by the next person for continuity of care.

**Objectives:**

The objective of this study was to investigate and describe the effects of increased nurses’ workload on quality documentation of patient information at PHC facilities in Vhembe District, Limpopo Province.

**Methods:**

The study was conducted in Vhembe District, Limpopo Province, where the effects of increased nurses’ workload on quality documentation of information is currently experienced. The research design was explorative, descriptive and contextual in nature. The population consisted of all nurses who work at PHC facilities in Vhembe District. Purposive sampling was used to select nurses and three professional nurses were sampled from each PHC facility. An in-depth face-to-face interview was used to collect data using an interview guide.

**Results:**

PHC facilities encountered several effects due to increased nurses’ workload where incomplete patient information is documented. Unavailability of patient information was observed, whilst some documented information was found to be illegible, inaccurate and incomplete.

**Conclusion:**

Documentation of information at PHC facilities is an evidence of effective communication amongst professional nurses. There should always be active follow-up and mentoring of the nurses’ documentation to ensure that information is accurately and fully documented in their respective facilities. Nurses find it difficult to cope with the increased workload associated with documenting patient information on the multiple records that are utilized at PHC facilities, leading to incomplete information. The number of nurses at facilities should be increased to reduce the increased workload.

## Introduction and background

Unavailability of the requisite staff members at PHC facilities increases nurses’ workload. Increased workload means nurses’ perform much more work than is normally required of them (Allen & Delahunty 2010:311). Nurses are expected to work effectively by rendering integrated multiple services daily as required by the Department of Health (DoH) in South Africa. Globally, the effect of increased workload due to staff shortages was felt acutely in both primary and secondary health care facilities (American College of Healthcare Executives 2012:03). Nurses experience heavy workloads in health care systems worldwide. Multiple documents are registers, books and forms for different programmes that are used at PHC facilities. A study that was done at eThekwini District, in KwaZulu-Natal, found that professional nurses at PHC facilities are faced with increased workload due to the daily integration of multiple PHC services, including new programmes being introduced (Vawda & Variawa 2012:09).

A study in a London hospital reported that the forms used in stoma care were partially completed by the nursing staff because these forms lacked standardization. The forms were not assessed during visits because multiple forms were used for one service. (Law, Akroyd & Burke 2010:07). An outcome of increased workload could increase patient mismanagement in both PHC and secondary facilities due to mistakes committed during the execution of multiple duties. Failure to document patient information leads to omissions of important data. If relevant information is omitted and any harm happens to a patient, relatives or next of kin may lodge complaints to the relevant authorities (South Africa 2006:07).

PHC facilities render 24-hour services in a call system, serving emergency patients; even the non-emergency cases visit the clinics for services as they know that there are always nurses after hours to render services. According to the *South African Basic Conditions of Employment Act* No 11 (South Africa 2015:08), as amended, an employer may not allow an employee to work for more than nine hours per day or for more than 45 hours per week. Pullen and Loudon (2006:280) reported that some professional nurses write patients’ notes long after an event had occurred due to increased workload.

Professional nurses reported that numerous factors contribute to increased workload at their respective facilities which lead to incorrect, illegible and inaccurate information being documented. According to the American College of Healthcare Executives (2012:03), in many countries around the world professionals were unable to cope with the growing mass of unmanaged records due to insufficient financial and administrative resources. Additionally, Joubert and Gerber (2011:10) argued that knowledge is an important tool for the professional nurses’ daily work of documenting services to patients and for the development of the organisation’s evidence base. Lack of the relevant knowledge at the workplace affects the efficiency and effectiveness of service delivery at facilities.

The growing complexity of programme forms and registers necessitates relevant staff with sufficient stationery in order to undertake the duties required. Rockville (2008:04) added that professional nurses were experiencing heavier workloads than before due to increased demand of such professionals in the health care systems in the face of reduced staffing and increased overtime. The issue of nurses being overworked has become a crisis throughout the world; even the local South African DoH is fully aware of nurses being overworked through working long hours per day and night to manage their workload.

The literature clearly indicates that professional nurses must be knowledgeable about the reasons for the availability of recorded information as the PHC facilities are the entry points of patient information. Patients visit PHC facilities before they are referred to a hospital with a referral letter, which should be readable so that the receiving professional nurses will be able to understand what is to be done to assist the patients (South Africa 2012:01).

Findings in the study done by Pullen and Loudon (2006:280) also indicated that health professionals do not share information with one another as they had a tendency to treat patient records as a personal record rather than a corporate asset, and hence little priority is given to records management by health professionals. Adding to the above findings, Meyer *et al*. (2011:106) indicated that there is a need for the required number of professional nurses to facilitate a proper integration of services so that nurses’ workload is reduced and adverse effects avoided.

An overworked nurse might fail to maintain quality documentation of patient information or even use the wrong stationery or misplace the completed documents leading to poor recording. Hence, the *South African Basic Conditions of Employment Act* No 11 (South Africa 2015:08), as amended, indicates that an employer might not allow an employee to work for more than the stipulated 9 hours per day or 45 hours per week. At PHC facilities, two nurses who work during the night (taking calls) were also expected to work during the day which increased their working hours; even though they are paid, they still complain of being tired.

The outcome of increased health professionals’ working hours could increase patient mortality and morbidity in both primary and secondary facilities (University of Maryland 2012–2013). Nurses’ working hours might be increased to compensate decreasing physician working hours in hospitals because professional medical associations have taken steps to limit their working hours (University of Maryland 2012–2013). At PHC facilities in Vhembe District nurses were experiencing increased working hours as there was a shortage of physicians at referral hospitals. Both the studies conducted in different facilities yielded similar results on the duration of working hours as a point of concern. Pullen and Loudon (2006:280) noted that nursing documentation was inadequate and incomplete because of the shortage of nurses to perform the work. Postponing documentation of patient information until after the event has occurred might lead to medico-legal hazards. Entry of patient information on records should be done immediately after an event has occurred to avoid forgetting important points and omitting information (Meyer *et al*. 2011:330).

### Problem statement

Generally, nurses in PHC facilities in Vhembe District experience undue pressure with increased workloads. An example is where one person is expected to do multiple duties such as caring for patients and documenting information. There were multiple registers and books at PHC facilities in which a patient’s information had to be recorded by one nurse for services rendered during a single visit. Patients coming for the first antenatal visit needed to be recorded in the tick register, PMTCT register and consent form for HIV and AIDS testing, HCT register, TB suspect register, blood specimen register, and maternity case record book. A BANC checklist also had to be completed. In Kenya, in three regions, nurses experienced increased workload due to shortage of staff, insufficient training and as a result of staff movement from government to private institutions for better salaries (Ojakaa, Olango & Jarvis 2014:07). According to the American College of Healthcare Executives (2014:03) in many countries around the world nurses were unable to cope with the growing mass of unmanaged records due to insufficient financial and administrative resources.

Increased workload due to the provision of free health services and the shortage of nurses were found to be the main problems with the provision of effective PHC services in all municipalities in Limpopo Province (Baloyi 2009:47). In Vhembe District, Limpopo Province, a research study on nursing documentation was performed by Rampfumedzi (2006:08) titled ‘Quality control of obstetric nursing records in a selected Regional Hospital’. The study focused on auditing obstetric nursing records. However, no participants were included. The findings of the study showed that the patient records were partially completed. Rampfumedzi (2006:08) further indicated that some information in the patient documents was not recorded at all, even though space to record information was provided and that no document was 100% completed.

#### Purpose of the study

The purpose of the study was to identify effects of increased nurses’ workload on quality documentation of patient information at selected PHC facilities in Vhembe District, Limpopo Province.

#### Research objectives

The objective of the study was to investigate and describe effects of increased nurses’ workload on quality documentation of information at PHC facilities in Vhembe District, Limpopo Province.

## Method and design

In this study, a qualitative, explorative, descriptive and contextual design was used (De Vos *et al*. 2011:269). The design enabled the investigator to explore and describe the effects of increased nurses’ workload on quality documentation of patient information. The views about effects of increased nurses’ workload on quality documentation of information were sought from professional nurses and described by the investigator.

### Data collection method

De Vos *et al*. (2011:335) defined data collection as a way of gathering information through asking questions, observations, voice recordings and taking field notes to answer rising research questions. The researcher used in-depth face-to-face interviews to collect different kinds of information from participants (Maree 2010:87). According to Maree (2010:87) an in-depth face-to-face interview is a two-way communication in which the researcher askes participants questions in order to learn more about their ideas, opinions and beliefs about the topic being studied.

Polit and Beck (2008:372) defined an in-depth interview as a one-to-one dialogue between the interviewee and researcher, a method whereby the researcher asks participants questions without preconceived opinions regarding the phenomenon and specific content. The following central question was developed and asked during the interview:

Explain in your own words the effects of increased nurses’ workload on quality documentation of information at your facility?

This question was further translated into Venda (the language spoken at the research setting) for participants to understand the information needed by the researcher. During individual interviews to collect information from each participant, probing, paraphrasing and follow-up for clarity was done (Babbie & Mouton 2009:180; Creswell 2009:186; De Vos *et al*. 2011:335). The responses in local language were translated into English by the researcher as she speaks Venda fluently. A voice recorder was used to assist the researcher to store and retrieve interview data. Consent to use a voice recorder was obtained from the participants, and field notes were written during interviews. Privacy was ensured as the interviews were conducted in a separate room that was not in use at that time.

### Data analysis

Data analysis is a creative examination process of information, making sense of the findings and deriving meaning of the collected data during a research process. The researcher implemented Tech’s eight steps of qualitative data analysis (Creswell 2009:186; Tesch 1990). The researcher read all written versions of the original material and wrote down ideas that came to mind and then picked one short interesting document, reviewed it and asked herself the question, ‘what was that about?’ The researcher made a list of thoughts and wrote them in the margin and grouped similar topics together. The researcher listed topics taken from information gathered and wrote them as segments of text. Categories were reduced by grouping and drawing lines between them. Abbreviations for each category were then arranged alphabetically. Material that belonged to each category was collated in one place. The researcher revised records after initial data analysis from which themes were developed and member checking was performed to audit information collected from participants.

## Trustworthiness

Trustworthiness was the approach used to clarify the notion of objectivity as manifested in qualitative research (De Vos *et al*. 2011:419-421). Four principles were applied to ensure trustworthiness. These strategies included credibility, dependability, confirmability and transferability, according to Lincoln and Guba’s framework (1985) as cited in Polit and Beck (2008:539). Credibility was ensured by prolonged engagement of the researcher with the participants; confirmability was ensured through discussing the findings of the study with supervisors and peers who have knowledge about qualitative research (Babbie & Mouton 2009:277). Transferability of the research findings was not possible, as with most qualitative studies, as the study was performed only in the Vhembe District of Limpopo Province.

## Ethical considerations

Ethical clearance was obtained from the University of Venda Health, Safety and Research Ethics Committee. Permission to collect data was obtained from the DoH, Limpopo Province, Vhembe District, Management of Mutale Sub-District and operational managers of PHC facilities. Informed consent was obtained from the participants. Voluntary participation, anonymity and confidentiality were ensured.

### Demographics of the participants

Data were collected from 10 nurses aged 24–49 years working at PHC facilities. All professional nurses had 3–15 years of experience. Participants were trained in Nurse-Initiated Management of Antiretroviral Therapy (NIMART).

### Results

The researcher identified:

- One health care professional related theme: effects related to increased workload with supporting quotations.
- Two sub-themes: the unavailability of patient documents and illegible, inaccurate and incomplete patient information (Table 1).

**TABLE 1:** Presentation of the findings: Theme and sub-themes.

Theme	Sub-themes
1. Effects related to increased workload	1.1: Unavailability of patient documents 1.2: Illegible, inaccurate and incomplete information

*Source*: Authors own work

#### Theme 1: Effects related to increased workload

In this study, increased nurse workload denotes that one nurse was expected to do multiple duties as far as documentation of the information was concerned. Recording of information in different books increased professional nurses’ responsibilities and workload during working hours. There were multiple registers and books at PHC facilities in which a patient’s information had to be recorded by a health professional for different services provided during a visit. Patients reporting for an antenatal first visit needed to be recorded in the tick register, PMTCT register and HCT register, HIV and AIDS consent form, TB suspect register, blood specimen register and the maternity case record book. A BANC checklist had to be completed.

According to the Record Management Policy (South Africa 2006:06), quality nursing documentation of patient information was a collective responsibility of employees, and documents should be kept updated by employees at all times to ensure that they were complete, accurate and readable. Increased workload faced by professional nurses at PHC facilities contributed strongly to incomplete and illegible documents. Borglund and Oberg (2008:08) argued in their study titled ‘How are records used in organizations?’ that employees found it easy to retrieve previous documents in order to make informed decisions in their workplace, thereby solving problems quickly. Patients’ updated information is thus considered key to quick and reliable decision-making in the workplace.

The National Patient Safety Agency (2013:14) identified communication difficulties as major factors affecting patient outcomes, as nurses were unable to read and understand information written by a colleague in a patient file or booklet. Particular concerns included unclear and incomplete nursing documentation that showed there was no confidence in reporting patient information as cited by Casey and Wallis (2011:01). Nurses read patients’ previous records to continue giving and integrating patient care. They communicated by exchanging information stored in patient records, thus enhancing, promoting and maintaining patient safety (Wang, Hailey & Yu 2011:10).

Failure to document patient information leads to omission of important facts and contributes to incomplete documents in facilities. In cases where information is not recorded in the patient’s file and omissions occur and if the patient is harmed, the patient’s relatives or next of kin might charge the responsible nurse or the employer (South Africa 2006:07). When a nurse is found to have failed to record interventions and observations performed, she or he could be charged with negligence due to the inadequate or missing information in the record source. It was indicated that there were many mistakes in hospital documents in England and Wales due to increased nurse workload. Mistakes made were due to invisible and incomplete documentation of patient information. There was also an unavailability of documents (Gilson 2014: 444).

**Sub-Theme 1.1: Unavailability of patient documents:** Nurses indicated during the interviews that, due to lack of space to store the documents in use in the facilities, some of the documents were misplaced or lost in the old cupboards. There was no plan of disposing old documents at PHC facilities as some of the clinics were still in possession of documents that were used when the facilities started operating 10 years ago. This situation resulted in the misplacement of useful documents at the facilities as the same cupboards also stored old documents. Illegible and incomplete information in the documents were also cited by the participants as significant challenges at the facilities.

The participants explained that professional nurses failed to record clear, readable patient information due to added work, carelessness and, sometimes, fatigue. They explained that as they worked 24 hours daily in a call system, they may be overworked during the night and forget to document the relevant patient information in the correct form or file. They even articulated that blank spaces in the files, forms and registers were due to tiredness, forgetfulness and increased workload. These factors contributed significantly to the unavailability of patient information. Owing to overwork, someone may fail to answer queries when documented patient information is illegible and incomplete. They also elaborated that some professional nurses had the tendency to record information later, after the incident had occurred and the patient had left the facility. This tendency leads to missing relevant information on how patient care was rendered. Nurses complained that, for a long time, they had reported the necessity of proper staffing to overcome the problems experienced with increased workload. The increased workload results in unavailable, illegible and incomplete documents.

Trained nurses at their workplaces have the ability to identify the challenges they encounter and how to resolve them, but the workload is increased as new programmes are introduced daily. Participants explained during interviews that, due to being overworked, they tended to write illegible information whilst trying to cover the workload. They even explained that some documents had gone missing because of lack of space to store them for later retrieval. Some of the required documents were stored in old cupboards due to limited space.

Participant 06, female age 44, general nursing and midwifery, 8 years’ experience and NIMART-trained said:

‘Another point is space where we keep our documents; clinics in rural areas have no adequate buildings. Sometimes we walk from one place to another to fetch patients’ documents. Due to such lack of space to keep documents and shortage of staff as there are no administrative clerks to assist in keeping documents we end up misplacing documents in the cubicles. We as nurses do not have time to file documents as we are always busy taking care of patients.’

Whereas Participant 09, male age 49, general nursing and midwifery, 6 years’ experience and NIMART-trained said:

‘No! Somewhere in the facility I mean in the postnatal ward which is also congested with equipments and treatment in use. There is no enough space; cubicles are very small and untidy as they are congested with books and equipments in use; wherein some of the documents got misplaced. When patients’ documents are missing or misplaced in facility it becomes difficult for a nurse to care for patients in totality as there would be no continuity of care. These also delay patient’s referral to other level of care and compromise care of patients.’

Participant 01, male age 24, BCUR degree, 3 years’ experience and NIMART-trained said:

‘According to the Department of Health an employee must work 8 hours per day and 40 hours per week regarded as normal working hours. Two nurses who work during the night [*taking calls*] are also expected to work during the day which increases their working hours, even though paid we get tired. When one has overworked during the night may forget to document required relevant patient’s information on the correct form or file and omit important information which is an evidence of what has happened.’

Singh and Seema (2014:04) confirmed in their study ‘Medical record department: An analytical study’ that serious limitations included the need for large storage areas for manual documents at health facilities because professional nurses experienced difficulties in the retrieval of documents in use. The failure of the officials to find the documents contributed more to unwritten documents, losing much-needed information. According to the Nursing and Midwifery Council (2013:07), disposal and retention of old documents at facilities depended on the policies and legislation of the government health policies of the country in which people practise. It is the duty of such departments to inform the employees about what might be done in relation to retaining and disposing such documents.

Even in Jordanian hospitals in the Middle East, the results of a study done on nursing documentation were the same as those of hospitals in England and Wales where documented patient information was invisible, incomplete and thus unavailable (Gilson 2014:444). According to a research study conducted by Pullen and Loudon (2006:07) in the United Kingdom, nursing documentation was accorded low priority. Documents were poorly maintained and some of the required records were not available in facilities. Furthermore, the authors explained that standards of nursing documentation were criticised by public bodies and in official inquiries into deficiencies of care in hospitals. The above findings indicate that, globally, more research on quality nursing documentation in public hospitals and PHC facilities needs to be done.

Moreover, in the study undertaken by Borglund and Oberg (2008:07) on ‘how are records used in organizations’ the employees are required demonstrate their ability to manage their work effectively − no information in documents should be missing. The primary aim of quality nursing documentation of patient information is that it is an essential source of information required for research studies. Unavailable, illegible and incomplete patient information made it difficult for researchers to identify the adverse events as there was insufficient data. Inadequate documented patient information in the records might indicate compromised patient care at the facilities concerned. The results of the study titled ‘Quality of patient record keeping: An indicator of the quality of care?’ by Zegers *et al*. (2011:02) revealed that there were missing components of documented patient information in both the nursing and medical records. Nursing documentation was found to be inadequate, illegible, incomplete, unclear and in a disorderly manner.

Participant 10, male, age 47, general nursing and midwifery, 15 years’ experience and NIMART-trained said:

‘According to the Department of Health an employee must work 8 hours per day and 40 hours per week regarded as normal working hours. Two nurses who work during the night [*taking calls*] are also expected to work during the day which increases their working hours, even though paid we get tired. When one has overworked during the night may forget to document required relevant patient s’ information on the correct form or file. These lead to unavailability of patient information in the available document which hinders proper continuity of patient care by the next professional nurse. That will frustrate both the nurse as the patient will be saying I visited the clinic last month but there is no documented information when the nurse check the document.’

Participant 09, male, age 49, general nursing and midwifery, 6 years’ experience and NIMART-trained said:

‘Because of lack of space to keep patient files, books, registers and other documents some of the required documents are imbedded in the old cupboards due to limited space to keep them accordingly. It also frustrates a nurse to search for patient’s document for a long time while he/she is waiting for the service to be rendered on the other hand patients waiting time is increased. Patients’ satisfaction is also affected and the results are poor services rendered to the community members. This will also increase a chance for patients to complain all the times about the services rendered.’

Participant 01, male, age 24, BCUR degree, 3 years’ experience and NIMART-trained said:

‘The burnout following multiple services at PHC facilities makes it difficult for us to perform daily duties and we do not meet the targets set by different programme managers, e.g., each facility has its own target for sputum collected per month which is two percent of headcount above five years and each nurse must counsel and test three clients daily for HIV/AIDS. As nurses we will just be saying we are working and very busy but the required information is not available as the targets are not reached.’

Participant 02, female, age 44, general nursing and midwifery, 5 years experience and NIMART-trained said:

‘Employees sometimes record information which they do not understand or fail to record information because they are not sure about what is to be recorded. These render facility documents to be incomplete and inaccurate thereby hindering informed decision making for the following nurse who will come across that document to continue rendering patient care. Then patient care will be disturbed as this official will fail to understand patient’s information documented by the previous nurse.’

Participant 04, male, age 42, general nursing and midwifery, 10 years’ experience and trained on NIMART and PHC said:

‘I think that it is professional nurses’ attitude towards quality documentation of patient information, as others display failure to ask from those informed about information to be recorded due to undermining others. Sometimes nurses do not write required information due forgetfulness. These really affect our facility patient documents and monthly statistics which is sent to the Vhembe District and that also affect facility performance and the plans of facility supplies and maintenance by DoH’.

Nurses exchange information stored in patients’ records thereby enhancing, promoting and maintaining patients’ safety. Continuity of service and informed decision-making is enabled when a nurse refers to the notes documented by a previous nurse (Wang, Hailey & Yu 2011:10). Moreover, in the study performed by Borglund and Oberg (2008:07) titled ‘How are records used in organizations’ the employees are required to demonstrate that they are able to manage their work effectively; no information in documents should be missing. The primary aim of quality nursing documentation of patient information is that it is an essential source of information required for research studies.

## Discussion

The study sought to investigate and describe the effect of increased nurses’ workload on quality nursing documentation of patient information at PHC facilities. The theme that emerged was the effects related to increased workload, and the sub-themes were the unavailability of patient information and documents, and illegible, inaccurate and incomplete patient information. The study has shown that increased nurses’ workload poses a serious challenge to the documented patient information and the documents themselves. To achieve sustained quality nursing documentation of patient information, changes in the PHC facilities are required, especially regarding increasing nursing staff and rationalising registers used for multiple programmes. Illegible, inaccurate and incomplete documented information may be the result of a shortage of manpower at facilities and thus the underlying cause why nurses fail to record information due to multiple duties. The more the documents are to be written by a few nurses executing the duty, the more they become demotivated and fail to record relevant information.

Studies have shown that nurses are faced with increased workloads. In this study, unavailability of information in some of the documents reported by nurses is the result of increased workload. Nevertheless, some professional nurses are trained in documenting information, but increased workload prevents them from keeping accurate documented information (Baloyi 2009:21). The literature indicated that there were some missing components of information in both nursing and medical records. Nursing documentation was found to be inadequate, illegible, incompletely understood and in disarray (Zegers *et al*. 2011:02). When there is a lot of work to be done by one person it makes one lose interest and fail to do what is expected, and more mistakes are made.

## Limitations of the study

There were challenges in the recruitment of the NIMART-trained nurses as some participants thought that the investigator wanted to find fault with the patient documented information in their respective facilities. The study was conducted at Mutale Local Area, Mutale Sub-District in Vhembe District, Limpopo Province. The study did not include nurses who were not NIMART-trained and in other lower categories. As with most qualitative studies where transferability is not feasible, in this study transferability of research findings is not possible as it was undertaken in one district of Limpopo Province.

## Recommendations

In order to minimise nurses’ workload at PHC facilities, complex changes are required. DoH executive authorities should assume responsibility to put a strategy in place for recruiting and strive to retain required nurses at facilities. With sufficient staff in the facilities, managers will allocate responsible staff to the different multiple programmes rendered at the facilities and where each staff member will be accountable for omissions of patient information in documents. This may reduce the effects of increased nurses’ workloads on quality nursing documentation of patient information.

Nurses who do not record the required information, even though they have been trained in the NIMART programme, must be confronted and assisted with the issues they do not understand. They may also be encouraged to work with others as a team, so that it is easy for them to ask others. Individual mentoring of nurses is important as it can bring good results as peoples’ challenges can be identified and dealt with.

Continuous mentoring and constant supervision by local area managers with relevant tools for all programmes can also improve the quality of documentation of patient information. Nurses as responsible DoH employees must be able to voice the challenges they come across at their workplaces and request assistance before they are charged with omission of important patient information. The teaching opportunities should also be used at workplaces by responsible managers as they can yield good results. Infrastructure should be modified and maintained continuously to enable nurses to execute their duties effectively.

## Conclusion

The presence of legible documents, accurate and complete information at PHC facilities provides evidence of effective communication amongst professional nurses, continuity of patient care, accountability and measuring of the facility’s performance. There should always be active follow-up and mentoring of professional nurses after they have attended workshops related to documentation of information at their respective facilities. Workshops are presented by programme managers, local area managers and NGO officers (responsible for data collection) at PHC facilities. This may be of great assistance and an eye-opener to those who are eager to learn. Tools should be available to assess all documents during monthly supervisory visits to facilities.

## References

[CIT0001] Allen R. & Delahunty A, 2010, *South African Oxford secondary school dictionary*, 2nd edn, University Press, Cape Town.

[CIT0002] American College of Healthcare Executives, 2014, *Ethical issues related to shortage of staff*, viewed 7 June 2014, from http://www.ache.org/abt-ache/socialresponsibility.cfm

[CIT0003] Babbie E. & Mouton J, 2009, *The practice of social research*, Oxford University Press, Cape Town.

[CIT0004] Baloyi L.F, 2009, *Problems in providing primary health care services: Limpopo province*, viewed 16 October 2014, from http://uir.unisa.ac.za/handle/10500/3131

[CIT0005] Borglund E.A.M. & Oberg L.N, 2008, ‘How are records used in an organization?’, *Information Research* 13(2), 1–18.

[CIT0006] Casey A. & Wallis A, 2011, ‘Effective communication: Principle of nursing practice’, *Nursing Standard* 25(32), 35–37. 10.7748/ns2011.04.25.32.35.c8450 21563538

[CIT0007] Creswell J.W, 2009, *Research design: Qualitative, quantitative and mixed methods approach*, Sage, London.

[CIT0008] De Vos A.S., Strydom H., Fouche C.B. & Delport C.L.S, 2011, *Research at grass roots for the social sciences and human service professions*, 4th edn, Van Schaik, Pretoria.

[CIT0009] Gilson L, 2014, *Implementation of new birth record in three hospitals in Jordan: A study in health system improvement*, viewed 28 February 2015, from http://www.who.int/countries/jor/en/10.1093/heapol/czm039 17965443

[CIT0010] Joubert H. & Gerber A, 2011, *Study guide: Introduction to diversity management*, Foundation of Professional Development, Lynwood Ridge, Pretoria.

[CIT0011] Law L., Akroyd K. & Burke L, 2010, ‘Improving nursing documentation and record keeping in stoma care, in London hospital’, *British Journal of Nursing* 19(21), 1328–1332, viewed 28 February 2015, from http://www.gala.greg.ac.uk/view/typesarticle.html 21355356 10.12968/bjon.2010.19.21.80002

[CIT0012] Maree K, 2010, *First steps in research*, 2nd edn, Van Schaik, Pretoria.

[CIT0013] Meyer S.M., Naude M., Shangase N.C. & Van Niekerk S.E, 2011, *The nursing unit manager: A comprehensive guide*, 3rd edn, Heinemann, Johannesburg.

[CIT0014] National Patient Safety Agency, 2013, *Patient safety alert*, viewed 10 September 2015, from http://www.nrls.npsa.nhs.uk/

[CIT0015] Nursing and Midwifery Council, 2013, *Record keeping 1–9*, viewed 4 April 2014, from http://www.connectingforhealth.nhs.uk

[CIT0016] Ojakaa D., Olango S. & Jarvis J, 2014, *Factors affecting motivation and retention of primary health care workers in three disparate regions in Kenya*, viewed 10 July 2015, from http://www.human-resources health.com/content/12/1/33 10.1186/1478-4491-12-33 PMC4097093 24906964

[CIT0017] Polit D.E. & Beck C.T, 2008, *Nursing research: Generating and assessing evidence for nursing practice*, 8th edn, Lippincott, Philadelphia, PA.

[CIT0018] Pullen J.I. & Loudon J, 2006, ‘Improving standards in clinical record-keeping’, *Advances in Psychiatric Treatment* 12(4), 280–286.

[CIT0019] Rampfumedzi D.P, 2006, ‘Quality control of obstetric nursing records in a selected regional hospital in Vhembe District in the Limpopo Province’, MA Health Studies thesis, University of South Africa, Pretoria.

[CIT0020] Rockville M.D, 2008, *Agency for healthcare research and quality*, viewed 21 February 2016, from http://www.ahrp.gov/research/findings/factsheets/children/publications/2008.htm 10.1080/15360280802537332 21923316

[CIT0021] Singh S. & Seema S, 2014, ‘Medical record department: An analytical study’, *International Journal of Management* 5(1), 14–22.

[CIT0022] South Africa: Department of Health, 2012, *District health management information system (DHIMS) standard operating procedures: Facility level*, Department of Health, Pretoria.

[CIT0023] South Africa: Department of Health and Social Development, 2006, *Records management policy*, Provincial Government Information and Records Management, Polokwane.

[CIT0024] South Africa: Department of Labour, 2015, *Basic conditions of Employment Act, 75 of 1997 and regulations as amended by Basic conditions of Employment, Act No 11 of 2015*, viewed 22 August 2015, from http://www.labour.gov.za

[CIT0025] Tesch R, 1990, *Qualitative research: Analysis types and software tools*, Falmer Press, New York.

[CIT0026] University of Maryland University College (UMUC), 2012–2013, *Catalogue: The Undergraduate School*, viewed 1 June 2014, from http://www.umuc.edu

[CIT0027] Vawda Y.A. & Variawa F, 2012, ‘Challenges confronting health care workers in government’s ARV rollout: Rights and responsibilities’, *Potchefstroomse Elektroniese Regsblad* 15(2), 487–519, viewed 10 February 2015, from 10.4314/pelj.v15i2.17

[CIT0028] Wang N., Hailey D. & Yu P, 2011, ‘Quality of nursing documentation and approaches to its evaluation: A mixed method systematic review’, *Journal of Advanced Nursing* 67(9), 1858–1875. 21466578 10.1111/j.1365-2648.2011.05634.x

[CIT0029] Zegers M., De Bruijne M., Spreeuwenburg P., Wager C., Groenewegen P.P. & Van Der Wal G, 2011, ‘Quality of patient record keeping: An indicator of the quality of care?’, *BJM Quality and Safety* 20(4), 314–318. 10.1136/bmjqs.2009.038976 21303769

